# Enhancing Concrete Mechanical Properties through Basalt Fibers and Calcium Sulfate Whiskers: Optimizing Compressive Strength, Elasticity, and Pore Structure

**DOI:** 10.3390/ma17071706

**Published:** 2024-04-08

**Authors:** Junzhi Zhang, Yueming Wang, Xixi Li, Yurong Zhang, Lingjie Wu

**Affiliations:** 1 College of Civil Engineering, Zhejiang University of Technology, Hangzhou 310014, China; jzzhang@zjut.edu.cn (J.Z.); 2112106057@zjut.edu.cn (Y.W.); 221122060130@zjut.edu.cn (X.L.); yrzhang@zjut.edu.cn (Y.Z.); 2 Key Laboratory of Civil Engineering Structure & Disaster Prevention and Mitigation Technology of Zhejiang Province, Hangzhou 310014, China; 3 College of Civil Engineering and Architecture, Wenzhou University, Wenzhou 325035, China; 4 Key Laboratory of Engineering and Technology for Soft Soil Foundation and Tideland Reclamation of Zhejiang Province, Wenzhou 325035, China

**Keywords:** calcium sulfate whiskers, basalt fibers, concrete compressive strength, dynamic elastic modulus, pore surface fractal dimension

## Abstract

To study the effects of basalt fibers (BFs), calcium sulfate whiskers (CSWs), and modified calcium sulfate whiskers (MCSWs) on the compressive strength and dynamic modulus of elasticity of concrete, this paper utilizes Mercury Intrusion Porosimetry (MIP) to measure the microstructure of concrete and calculate the fractal dimension of pore surface area. The results indicate that both CSWs and BFs can increase the compressive strength of concrete. CSWs can enhance the dynamic modulus of elasticity of concrete, while the effect of BFs on the dynamic modulus of elasticity is not significant. The improvement in compressive strength and dynamic modulus of elasticity provided by MCSWs is significantly greater than that provided by CSWs. Both CSWs and BFs can effectively improve the pore structure of concrete and have a significant impact on the surface fractal dimension. CSWs inhibit the formation of ink-bottle pores, while BFs increase the number of ink-bottle pores. Due to the ink-bottle pore effect, the fractal dimension of the capillary pore surface is generally greater than three, lacking fractal characteristics. The compressive strength and dynamic modulus of elasticity of concrete have a good correlation with the fractal dimensions of large pores and transition pores.

## 1. Introduction

Concrete, a heterogeneous and multiphase composite, develops pores and cracks as it hardens—integral elements of its internal architecture. The mechanical attributes of concrete are pivotal for evaluating its overall performance, and these internal structures have a significant impact on these properties [1,2]. With advancements in material science and technology, concrete’s enhancement in terms of weight reduction and improved strength continues. The integration of assorted materials to boost its performance is a primary research focus. Fibrous materials enhance inter-component cohesion and contribute to the formation of additional calcium silicate hydrate (CSH) gels through hydration, refining the pore network. Similarly, nanomaterials affect the gel’s morphology, improving the pore structure [3,4,5,6].

Fibers, especially, have been shown to bolster concrete’s fundamental properties by reinforcing bonds between its constituents [3,7,8]. Basalt fibers (BFs), derived from basalt rock, are prized for their high tensile strength, stability, and resistance to corrosion. These properties align well with cementitious materials, prompting their increased application in concrete [9]. Research by Fang et al. [10] demonstrates the influence of BF content on concrete’s compressive strength, proposing a predictive model based on neural network analysis. Sun et al. [11] observed that compressive strength initially increases with BF content, then diminishes. Al-Rousan et al. [12] confirmed that an optimal BF quantity elevates concrete’s dynamic modulus of elasticity, albeit a surplus can reduce it.

Nanoparticles, with their extensive surface area relative to volume, exhibit unique properties distinct from conventional solids—size, quantum, surface, and interface effects [13,14]. These facilitate pore structure refinement in concrete [15,16]. Calcium sulphate whiskers (CSWs), a hybrid of fibers and nanoparticles, possess the granularity of fillers and the aspect ratio of fibers, providing dimensional stability, tensile strength, toughness, heat resistance, and chemical durability [17,18,19], making them suitable for cement-based applications. Eteläaho et al. [20] found CSWs to incrementally raise concrete’s compressive strength, enhancing bond quality at the cement matrix interface, thus reinforcing and toughening the composite. Turkmenoglu et al. [21] observed that nanomaterials raise the dynamic modulus of elasticity in concrete, with the nano-filling effect homogenizing its microstructure. Chen et al. [22] noted that CSWs activate fly ash, promoting ettringite formation in early stages, mitigating crack development, and enhancing early strength in fly ash concrete. As nanomaterials, CSWs can fill internal voids, and as fibrous materials, they manage microcracking, thus diminishing porosity and strengthening base mechanical properties.

The high surface free energy of needle-like CSWs compared to lower energy in composite materials poses compatibility challenges, potentially diminishing performance [20]. Surface modifications, such as hydrophobic treatments, are often employed to improve CSW interactions within the concrete [23,24,25]. Studies by Wang [26], Liu [23], and others have shown that whisker treatment with coupling agents creates stable chemisorption layers, enhancing interfacial bonding. Zhang et al. [27] found that modifiers could expedite the hydration process, generating more CSH gel and densifying the material structurally.

BFs and CSWs directly affect the mechanical properties and durability of concrete, among other macroscopic properties, by altering the pore structure of the concrete [28,29,30,31]. However, the pore structure of concrete is complex and disordered, making it difficult for traditional parameters such as porosity to fully describe its complexity [30]. Fractal theory, which examines the irregular geometric patterns of fractal materials, is apt for concrete’s irregular morphology, exhibiting a spectrum of fractal characteristics [31]. Various models, including spatial, Menger sponge, and thermodynamic models, have been employed to analyze concrete’s fractal nature [32,33]. Despite the Menger sponge model’s detailed multi-level pore simulation, its idealized structure yields inaccuracies in fractal dimension calculations. Zhang’s thermodynamic fractal model aligns more closely with actual pore conditions, leveraging MIP test functions to produce reliable and precise pore characteristic descriptions [31,32]. Hence, this model is preferred for characterizing concrete’s pore structure in numerous studies [32].

This paper investigates the influence of CSWs and BFs on the compressive strength and dynamic modulus of elasticity in concrete. We designed mixtures with varying CSW and BF concentrations, complemented by a modified calcium sulphate whisker (MCSW) variant. We analyzed the mechanical properties and microstructure through MIP, employing the thermodynamic fractal model to compute the surface fractal dimension—thereby assessing pore structure complexity. By correlating fractal dimensions with macro-properties, we aim to elucidate the effects of CSWs and BFs on concrete’s mechanical faculties and pore structure micro-characteristics.

## 2. Experimental

### 2.1. Raw Materials

The primary binder utilized in this study was a composite Portland cement, Conch brand P.C 42.5, characterized by a chemical composition of 67.71% CaO, 14.95% SiO_2_, and 7.96% Al_2_O_3_. The aggregates used in the concrete mix were sourced from the Hangzhou region, Zhejiang Province—ordinary river sand with a fineness modulus of 2.65 served as the fine aggregate, and continuously graded gravel served as the coarse aggregate, with an apparent density of 2600 kg/m^3^ and a maximum particle size of 20 mm. The water absorption rate of the coarse aggregate used in the experiment is 0.64%, while that of the fine aggregate is 1.09%.

**Figure 1:**
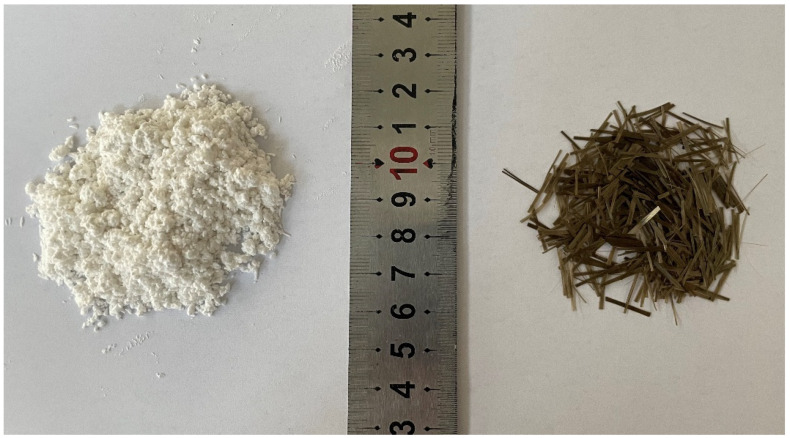
Physical diagram of CSW and BF.

**Figure 2:**
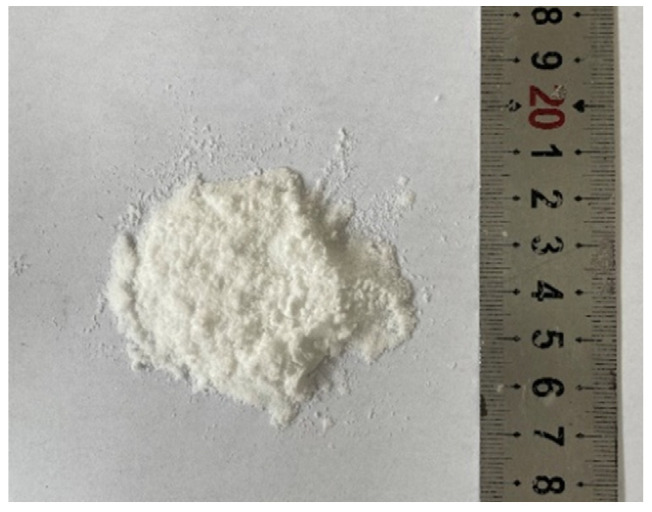
Physical diagram of SHMP.

**Figure 3:**
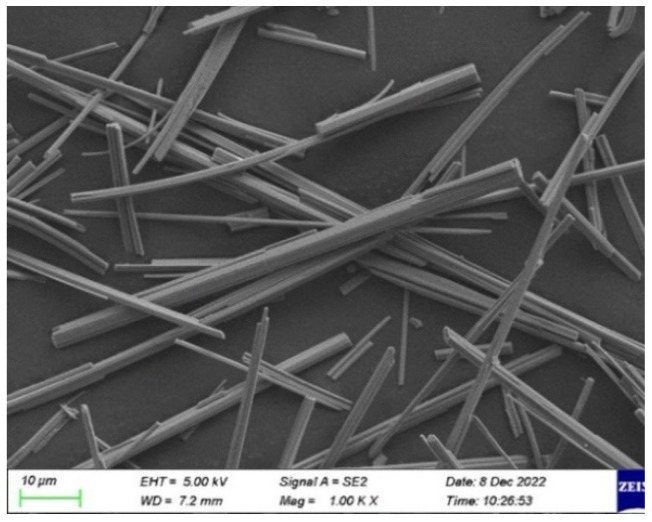
SEM image of the CSW.

The CSWs, manufactured by a Shanghai-based company (refer to Figure 1), exhibited an averaged aspect ratio of 120. The surfactant used for the CSW was sodium hexametaphosphate (SHMP), depicted in Figure 2. The primary performance parameters of the CSW are summarized in Table 1, and their scanning electron microscopy (SEM) image is provided in Figure 3. Short-cut BFs, produced by a Zhejiang Province fiber joint-stock company (illustrated in Figure 1), had their key performance metrics detailed in Table 2.

### 2.2. Specimen Preparations

Guided by the “Hydraulic Concrete Test Procedure” (SL/T 352-2020) [34], this study devised two concrete batch types with water–cement ratios (*w*/*b*) of 0.40 and 0.50. Each *w*/*b* ratio category included one ordinary concrete (OC) set, four CSW concrete sets (CC1~CC4), four BF concrete sets (BFC1~BFC4), and one MCSW concrete set (SCC), with a total of twenty mix sets. The concrete mix ratios are itemized in Table 3.

For specimen fabrication, following SL/T 352-2020 [34], we constructed three cubes with side lengths of 150 mm for each mix to assess compressive strength and three prisms of 100 mm × 100 mm × 400 mm to determine the dynamic modulus of elasticity. Additionally, three cubes with side lengths of 10 mm were excised from the prisms for MIP microstructural examination. The averages of the three specimens’ measurements were used for the dynamic modulus of elasticity, compressive strength, and microstructural parameter analyses.

The CSW modification Involved adding 1.5 g of SHMP and 3.0 g of CSW to 300 mL of deionized water, stirring thoroughly, then heating at 60 °C for 2 h in a water bath. Post-heating, the CSW was oven-dried. During specimen mixing, to enhance BF and CSW dispersion, aggregates, cement, and fibers were first blended under the dry condition in a forced mixer for 2 min. The CSW was then dissolved in water and ultrasonically dispersed for 5 min at 500 W. This solution, along with additional water, was added to the mixer for an additional 2 min of wet blending. This protocol was designed to ensure uniform dispersion of fibers and nanoparticles without clumping.

### 2.3. MIP Testing Method

The concrete pore structure was analyzed using an AUTOPORE 9500 mercury intrusion porosimeter, accommodating both high- and low-pressure assessments with intrusion pressures up to 33,000 psi and a pore size detection range between 0.005 and 1000 μm. Pre-test, samples were desiccated at 105 °C for 48 h to eliminate internal moisture. Each sample’s mass was measured with 0.001 g precision before and after expansion agent application. Following the low-pressure assessment, the sample underwent high-pressure testing.

Mercury intrusion pressures were determined using the Washburn–Laplace equation [35]:

(1) P=2γcosθ/R

where *P* is the intrusion pressure (Pa); *θ* the contact angle between mercury and sample; *γ* the mercury’s surface tension (N/m); and *R* the pore radius (m).

### 2.4. Fractal Dimension Model

The fractal dimension was calculated using Zhang’s thermodynamic model [33], which posits that the work by external force on mercury equates the mercury liquid interface’s surface energy:

(2) dW=−PdV=γcosθdS

where *V* represents the intrusion volume of mercury (m^3^) and *S* denotes the surface area of the pore (m^2^).

The pore surface area *S* is expressed as a function of the radius *R*_n_ and cumulative intrusion volume *V*_n_, where k is the related coefficient and *D*_s_ the fractal dimension of the pore surface [33]:

(3) $${\left(S\right)}_{n}=k^{D_{s}}R_{n}^{2-D_{s}}V_{n}^{D_{s}/3}$$

Substituting Equation (3) into Equation (2) and rearranging, followed by integration and logarithmic transformation, yields

(4) $$\mathrm{In}\left(\frac{\sum_{i=1}^{n}P_{i}\Delta V_{i}}{R_{n}^{2}}\right)=D_{s}\cdot \mathrm{In}\left(\frac{V_{n}^{1/3}}{R_{n}}\right)+C$$

where *C* is a constant.

The slope attained from linear regression in Equation (4) determines the fractal dimension *D*_s_, indicative of the pore surface’s complexity.

## 3. Results and Discussion

### 3.1. Compressive Strength

**Figure 4:**
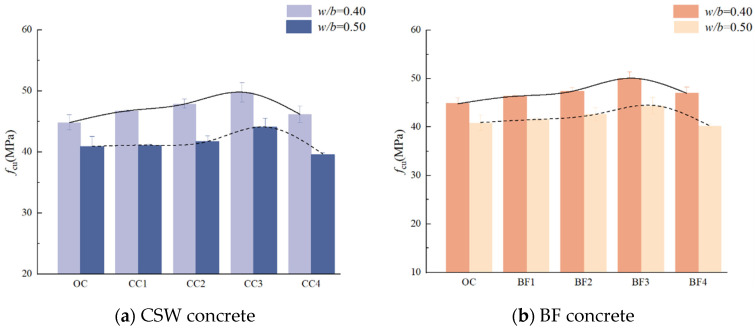
Compressive strength of CSW and BF concrete.

Figure 4 delineates the compressive strength variation in concrete specimens incorporated with CSW and BF at different *w*/*b* ratios. It is apparent from the figure that a lower *w*/*b* ratio (0.40) significantly enhances the compressive strength compared to a higher *w*/*b* ratio (0.50). This is because, as the *w*/*b* ratio increases, the content of C-S-H and Ca(OH)_2_ inside the concrete decreases, resulting in more pores. Conversely, a lower *w*/*b* ratio can improve the interfacial transition zone of the concrete, reducing the number and size of internal pores [36].

This study reveals that the CSW and BF content profoundly influences concrete’s compressive strength. An increasing trend in strength is observed with the addition of CSW up to 4.5 kg/m^3^, which represents the optimal content for both *w*/*b* ratios. Beyond this content, a decline in strength is noted, a phenomenon attributed to the augmented porosity and emergence of interfacial defects between the whiskers and cement paste [21,23]. When the dosage of BF ranges from 0 to 4.5 kg/m^3^, the compressive strength of concrete also increases with the increase in BF content, and the compressive strength of concrete with both *w*/*b* ratios reaches its maximum value at a dosage of 4.5 kg/m^3^. BF has a high modulus of elasticity, and the appropriate addition of BF can form a randomly distributed support system in concrete, inhibiting the development of microcracks in the matrix, thereby enhancing the compressive strength of concrete [30]. However, when the BF content is too high, the fibers are unevenly dispersed, and the fibers fold and entangle with each other, forming weak points, which reduces the compressive strength of the concrete [37].

**Figure 5:**
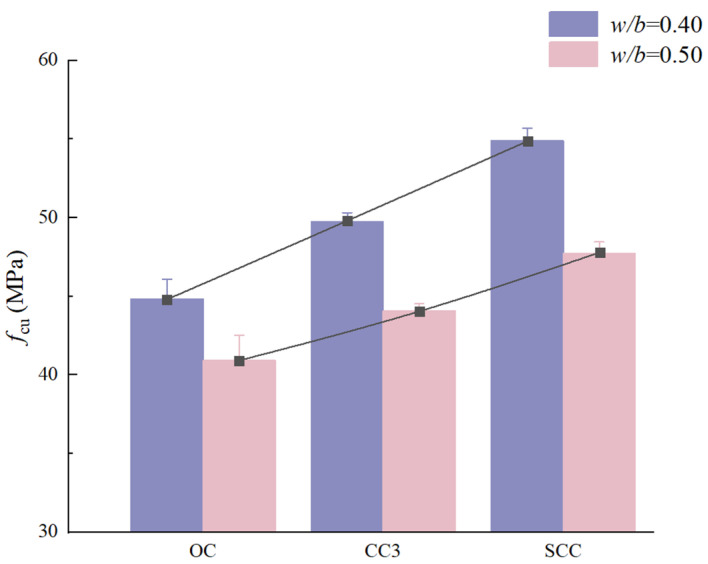
Compressive strength of CSW and MCSW concrete with different *w*/*b* ratios.

In comparison, as presented in Figure 5, concrete specimens with MCSWs showcase a considerable enhancement in compressive strength, registering increases of 10.33% and 7.61%, respectively, over their CSW counterparts. The superior performance of MCSWs is likely due to their smaller diameter and reduced incidence of breakage, resulting in more robust interfacial bonding with the matrix and a more homogeneous distribution within the concrete, thereby bolstering the mechanical attributes [27,38].

### 3.2. Dynamic Elastic Modulus

**Figure 6:**
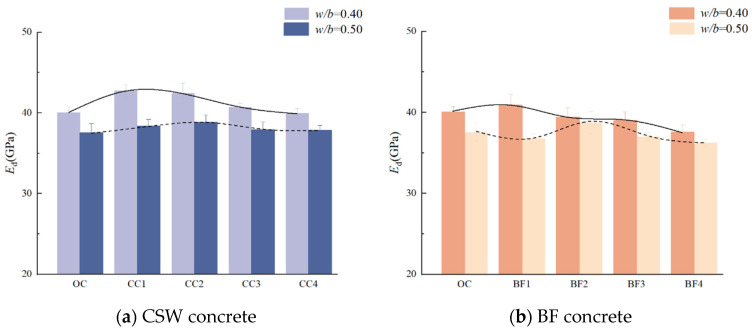
Dynamic elastic modulus of CSW and BF concrete.

**Figure 7:**
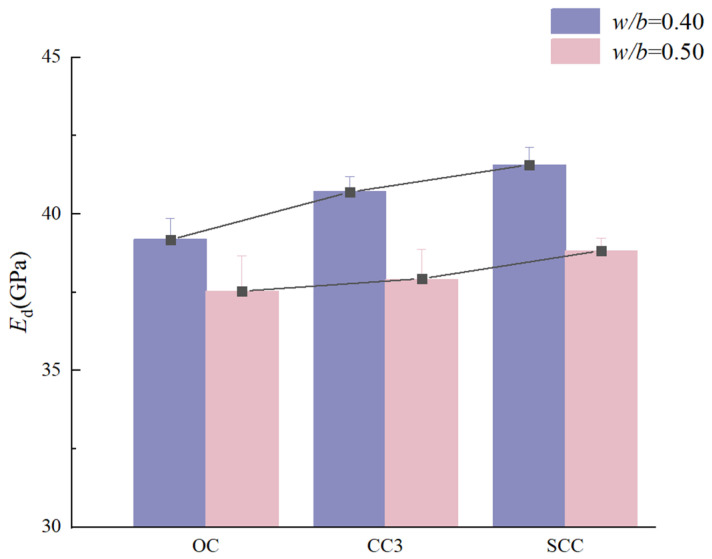
Dynamic elastic modulus of CSW and BF concrete with different *w*/*b* ratios.

Dynamic modulus of elasticity variations as a function of CSW and BF content are graphically represented in Figure 6. Corresponding tendencies for concrete with MCSWs in comparison to CSW concrete are depicted in Figure 7. An initial increase followed by a decrease in the dynamic modulus is discernible with rising CSW content. Notably, the optimal CSW content diverges between the two *w*/*b* ratios, being 1.5 kg/m^3^ for *w*/*b* 0.40 and 3.0 kg/m^3^ for *w*/*b* 0.50. The dynamic modulus demonstrates disparate trends with increasing BF content across the *w*/*b* ratios, with peak values for distinct BF content for each ratio. MCSW inclusion appears to marginally enhance the dynamic modulus of elasticity, though not to the same extent as the observed improvements in compressive strength.

### 3.3. Microstructural Parameters

MIP tests yield crucial parameters regarding the pore structure of concrete, such as porosity, median pore size, and average pore size [39,40]. These parameters are consolidated in Table 4 for CSW, MCSW, and BF modified concrete. A trend of decreasing total porosity, and median and averaged pore size, followed by an increasing trend, aligns with the escalation of CSW content. The least porosity and pore size metrics are observed at a CSW content of 3.0 kg/m^3^ for both *w*/*b* ratios. This is primarily due to the combined effects of micro-aggregate filling and fiber bridging. The micro-aggregates effectively fill the internal pores of the concrete, while the fiber materials bridge across cracks, inhibiting the formation of micro-cracks and controlling the further development of pore size [20]. Relative to OC, these modifications result in a significant reduction in porosity and pore size. MCSWs further enhance these reductions, suggesting substantial improvements in the microstructural quality of the concrete. BF content also follows an analogous trend, with optimal reductions for specific BF content based on the *w*/*b* ratio. Existing studies have shown that BF not only plays a role in bridging fibers to inhibit the occurrence of cracks in concrete, but the hydrophilic nature of BF also results in a higher *w*/*b* ratio in the concrete slurry surrounding the fibers, which promotes the hydration of the surrounding cementitious materials [41].

**Figure 8:**
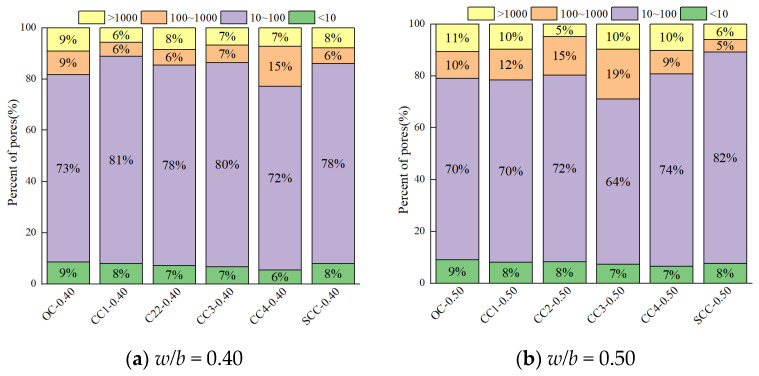
Pore size distribution of CSW concrete.

**Figure 9:**
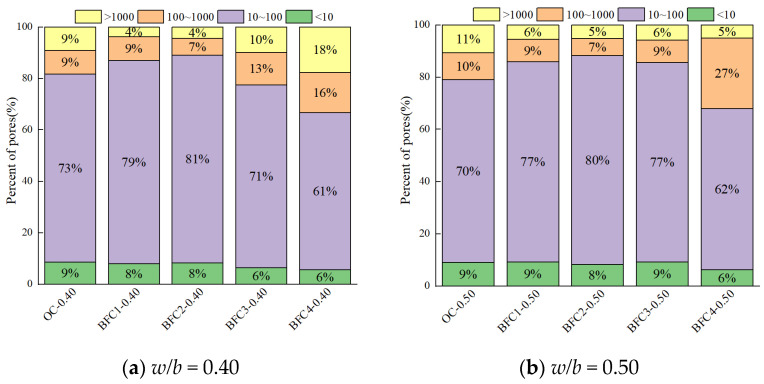
Pore size distribution of BF concrete.

Figure 8 and Figure 9 illustrate the pore size distribution, designating four distinct pore size regimes for analysis. Transitional pores, spanning 10–100 nm, predominate the pore size distribution, composing roughly 70% of the total porosity. The smallest fraction, gel pores less than 10 nm, contributes to about 8% of the porosity. The proportions of macropores and capillary pores are considerably smaller than transitional pores. A higher *w*/*b* ratio (0.50) markedly increases the proportions of macropores and capillary pores, implicating them as principal factors in the rise of total porosity [42].

In Figure 8, the proportion of gel pores remains fairly constant with increasing CSW content, while the combined proportion of macropores and capillary pores experiences significant variations. Excess CSWs intensify these larger pore fractions, implying a detrimental effect on the concrete’s structural integrity [21]. Conversely, MCSWs substantially curtail the proportion of these larger pores, indicative of their efficacy in improving concrete’s microstructure.

Figure 9 reveals that BF content does not significantly impact gel pore proportions; however, the combined proportion of larger pores initially decreases and then increases. Excessive BF addition poses dispersion challenges, leading to reduced bonding performance, enhanced weak interfaces, and a consequent increase in macropores, which adversely affects the concrete’s density and mechanical performance [30].

### 3.4. Pore Characteristics

**Figure 10:**
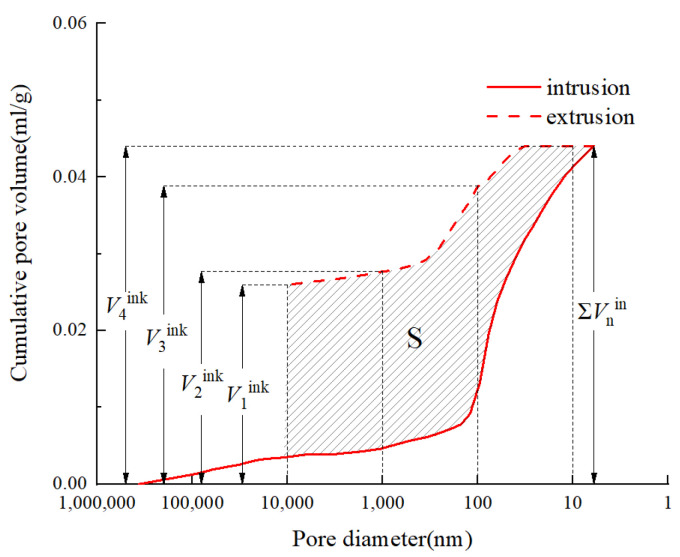
Pore size distribution from MIP testing.

Concrete encompasses various pore types, typically categorized into open and closed pores. Open pores are subclassified into fully and semi-open, with ink-bottle pores being a subcategory of semi-open pores characterized by incomplete mercury expulsion during MIP tests [43]. MIP identifies only open pores, the total volume of mercury intruded equating to the open pore volume. Ink-bottle pore presence is the primary cause for the discrepancy observed between intrusion and extrusion curves, as depicted in Figure 10, for a representative specimen of OC-0.40. This figure highlights the area between these curves, corresponding to the cumulative volume of ink-bottle pores.

The hysteresis loop area (S), enclosed by the mercury intrusion and extrusion curves, indicates the prevalence of ink-bottle pores within the concrete structure [44]. The cumulative volume of mercury intrusion, denoted by Σ*V*_n_^in^, and the ink-bottle pore volume, represented as *V*_n_^ink^, n = {1,2,3,4}, are computed for different pore size ranges utilizing Equations (5) and (6) [45]. Correspondingly, Δ*V*_n_^ink^, n = {1,2,3,4} reflects ink-bottle pore volumes across various pore sizes, including gel pores, transition pores, capillary pores, and macropores.

(5) $$\Delta V_{n}^{\mathrm{ink}}=V_{n+1}^{\mathrm{ink}}-V_{n}^{\mathrm{ink}},\;n=\left\{1,2,3\right\}$$

(6) $$\Delta V_{4}^{\mathrm{ink}}=\sum V_{n}^{\mathrm{in}}-V_{4}^{\mathrm{ink}}$$

Table 5 presents averaged values for the hysteresis loop area and ink-bottle pore volumes in distinct pore size ranges. The data elucidate that CSWs and BFs predominantly influence the volume of ink-bottle pores in the capillary and transition pore regions, with the highest concentration in capillary pores and negligible in gel pores. The hysteresis loop area generally diminishes with the addition of CSWs, while an increase in BFs correlates with a larger hysteresis loop area compared to ordinary concrete, suggesting greater ink-bottle pore generation [9,21]. A whisker or fiber content of 3.0 kg/m^3^ marks the smallest hysteresis loop area. Analysis of OC, CC3, and SCC asserts that MCSWs effectively decrease ink-bottle pore content, enhancing the microstructure’s integrity.

### 3.5. Surface Fractal Dimension

**Figure 11:**
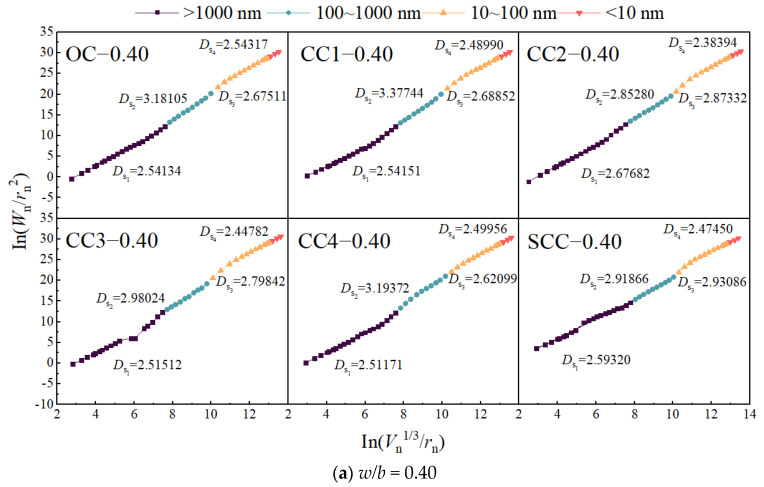

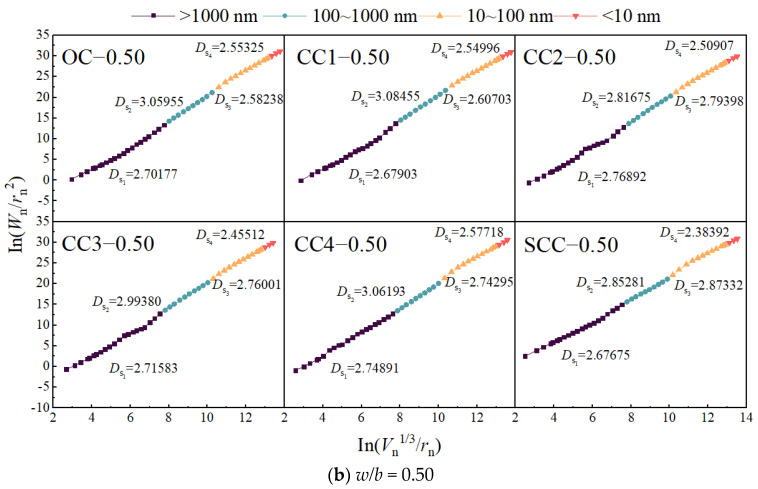
Pore surface fractal dimension curves of CSW concrete.

**Figure 12:**
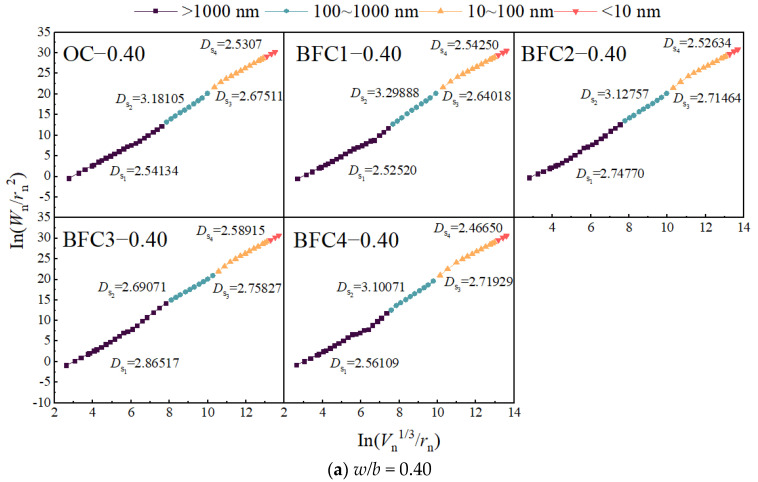

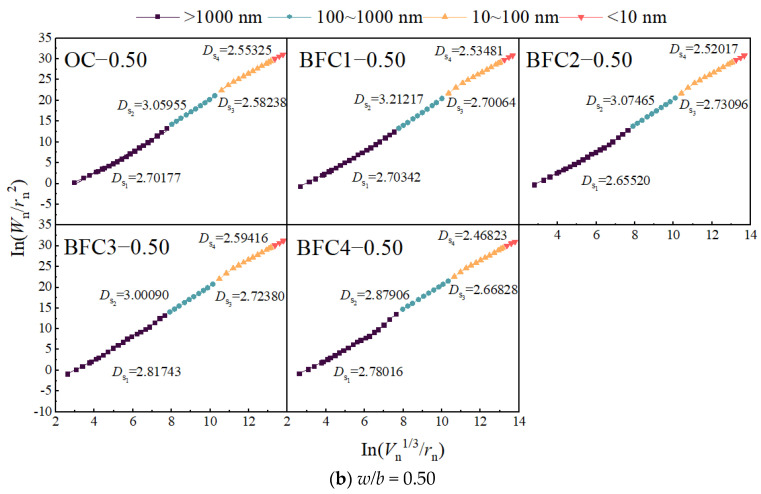
Pore surface fractal dimension curves of BF concrete.

*D*_s_ embodies concrete’s internal pore structure and is deduced from the slope of an In-In plot crafted using Equation (4). This dimension reflects the fractal nature, size-dependence, and multifractal characteristics of concrete’s pore distribution and shape diversity [35,46]. Figure 11 and Figure 12 categorize pore characteristics by size: gel pores (<10 nm), transition pores (10–100 nm), capillary pores (100–1000 nm), and macropores (>1000 nm).

An examination of Figure 11 and Figure 12 indicates that the surface fractal dimension for macropores (*D*_s1_), transition pores (*D*_s3_), and gel pores (*D*_s4_) oscillates between two and three, indicating a range consistent with three-dimensional Euclidean space surface structures. Capillary pores (*D*_s2_) exhibit a larger fractal dimension, predominantly exceeding three, which lacks physical significance and thus does not conform to fractal behavior [33,44]. Singularities within the fractal dimension curve, marked by a sudden change and dimensions equal to or greater than three, are indicative of ink-bottle pore influence [45].

*D*_s_ is indicative of the intricacy of a porous surface, encompassing not just porosity and pore size, but the morphology of the pores as well. A higher *D*_s_ suggests a more complex pore structure. Upon scrutinizing Figure 11, the *D*_s_ of concrete laced with CSW presents a distinctive behavior: *D*_s1_ is markedly erratic; *D*_s3_ peaks at a whisker content of 3 kg/m^3^, indicating a transition from capillary to transitional pores, thereby maximizing the surface area and roughness at this content level; *D*_s4_ is notably reduced in comparison to ordinary concrete when the whisker content is 3 kg/m^3^. Nonetheless, the gel pores’ benign nature means they only marginally influence the concrete’s mechanical properties [21].

Concretes with MCSWs exhibit a *D*_s3_ substantially surpassing that of standard CSW concrete. This is attributable to the reaction of SHMP with the whiskers, forming an insoluble layer that mitigates whisker dissolution [23]. The even dispersion of these modified whiskers densifies the concrete structure and precludes the emergence of crystal flaws or cracks, consequently raising *D*_s3_ as larger pores are supplanted by innocuous micropores.

Figure 12 intimates that *D*_s1_ is at its apex when BF content reaches 4.5 kg/m^3^. The primary reason is that an excessive addition of fibers can lead to difficulties in dispersion, resulting in weakened bonding performance between the fibers and the concrete matrix. Moreover, it becomes easier to introduce air bubbles, thereby increasing the porosity, reducing the pore surface area, and decreasing *D*_s1_. This is consistent with the research findings of Li [30]. *D*_s3_ shows a rise followed by a decline, less pronounced than that observed with CSWs, signaling the latter’s more significant effect on the transitional pore area. BFs have a negligible influence on *D*_s4_, meaning that a minor impact on this pore region is suggested.

Comparison of Figure 11 and Figure 12 underscores that CSWs and MCSWs significantly affect the *D*_s3_ of concrete, whereas BFs chiefly influence *D*_s1_. This suggests that CSWs primarily enhance the pore structure by increasing pore roughness in the transition zone, while BFs modify the structure by increasing the roughness of larger pores. CSWs, acting as a microfiber and sub-nanometer filler, not only bridge hydration products, reinforce aggregate-matrix bonds, and curb micro-crack development but also promote hydration product consumption, leading to more CSH and ettringite formation, reducing pore connectivity, and thereby increasing the pore surface’s roughness and surface fractal dimension [47]. Conversely, BFs, functioning as macro fibers, form a stable network within concrete, inhibit macro crack development, and enhance roughness in the interfacial transition zone, thus elevating the surface fractal dimension [41].

Previous studies indicate that combining CSWs and fiber materials can synergistically optimize concrete’s pore structure [48]. CSWs and their product, CaSO_4_•2H_2_O, fill the voids between fibers and the matrix, further refining the pore architecture and optimizing pore size distribution. Thus, integrating CSWs and BFs into concrete and deeply analyzing their impact on pore structure roughness and their mechanism of action holds significant potential for improving concrete’s macroscopic performance.

**Figure 13:**
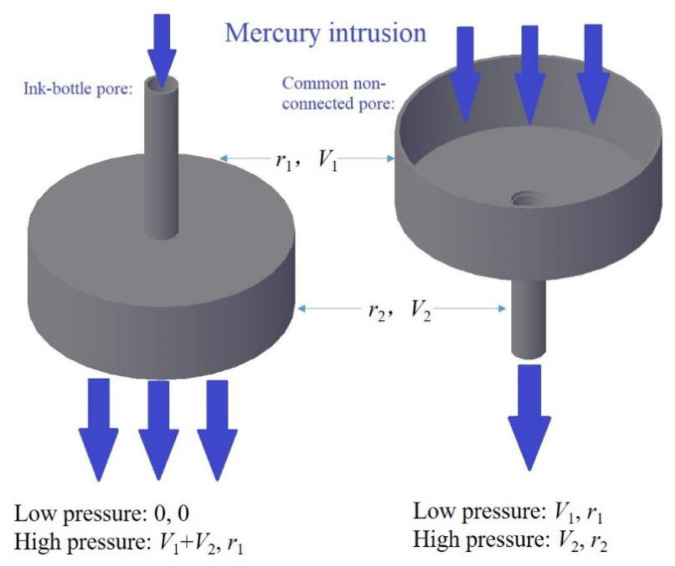
Simulated diagram of ink-bottle pores and regular non-connected pores.

Figure 13 schematically represents ink-bottle pores alongside typical non-communicating pores. The diagram illustrates the mercury intrusion direction, with *V*_1_ and *V*_2_ quantifying mercury volumes at varying pressures, and *r*_1_ and *r*_2_ signifying the corresponding pore sizes. MIP tests for conventional semi-open pores register a cumulative volume increase from *V*_1_ to *V*_1_+*V*_2_ and a pore size expansion from *r*_1_ to *r*_2_ with escalating mercury pressure. In contrast, ink-bottle pores necessitate sufficient mercury pressure to log the cumulative volume *V*_1_+*V*_2_ while only recording a pore size of *r*_1_ [30]. Consequently, ink-bottle pores tend to exhibit a larger cumulative mercury volume at pore size *r*_1_, raising the potential for surface fractal dimensions surpassing three.

### 3.6. Relationship of Surface Fractal Dimension with Compressive Strength and Dynamic Elastic Modulus

**Figure 14:**
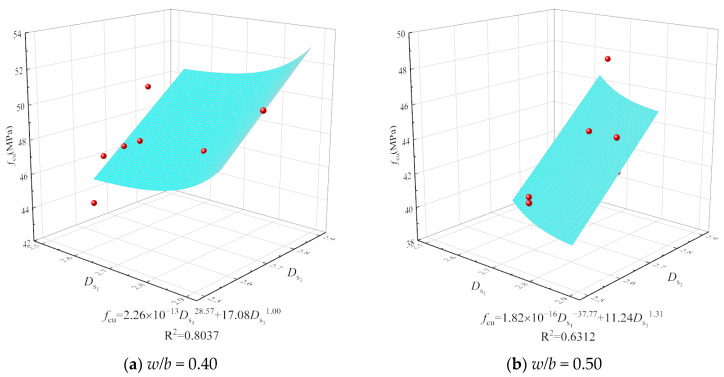
Relationship of compressive strength with *D*_s1_ and *D*_s3_.

**Figure 15:**
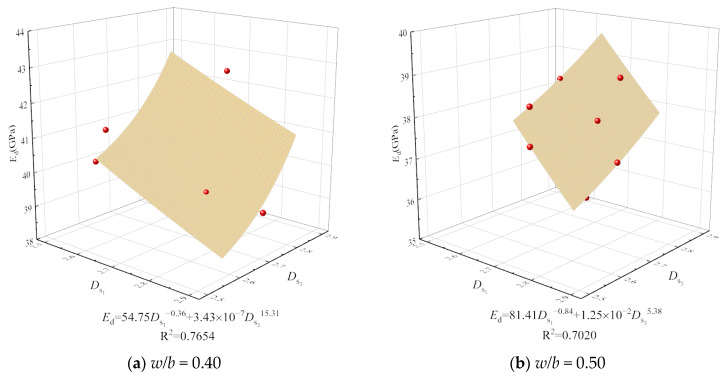
Relationship of dynamic elastic modulus with *D*_s1_ and *D*_s3_.

Given the optimal content variation for the mechanical properties of concrete with different mix ratios, assessing the influence of CSWs and BFs solely based on optimal content is insufficient. It necessitates a comprehensive micro–macro analysis, considering porosity, pore shape, size, surface area, and spatial distribution [31]. The surface fractal dimension crucially characterizes pore structure complexity. This study thus explores the correlation between the surface fractal dimension of concrete with CSWs and BFs and its dynamic modulus of elasticity and compressive strength, focusing on *D*_s1_ and *D*_s3_ due to *D*_s2_’s non-fractal nature and minimal gel pore impact. Figure 14 and Figure 15 delineate the correlations, which conform to a power function and display discernable associations.

It is observed from Figure 14 and Figure 15 that the relationships between compressive strength, dynamic elastic modulus, *D*_s1_, and *D*_s3_ approximately follow power functions, as shown in Equations (7) and (8), and exhibit certain correlations.

(7) $$f_{\mathrm{cu}}=a_{1}D_{s1}^{b_{1}}+c_{1}D_{s3}^{d_{1}}$$

(8) $$E_{d}=a_{2}D_{s1}^{b_{2}}+c_{2}D_{s3}^{d_{2}}$$

where *f*_cu_ represents the compressive strength of concrete in MPa; *E*_d_ represents the dynamic elastic modulus of concrete in GPa; and *a*_1_, *a*_2_, *b*_1_, *b*_2_, *c*_1_, c_2_, *d*_1_, and *d*_2_ represent regression coefficients.

The surface fractal dimension’s positive correlation with dynamic modulus of elasticity and compressive strength demonstrates that a higher fractal dimension equates to an enhanced ability to distribute stress within the concrete’s internal structure, uplifting compressive strength [49]. The dynamic modulus is affected by the microstructure and the properties of the aggregate and cement paste materials [50]. When partially connected pores are replaced by smaller pores, increasing the surface area, the surface fractal dimension also rises. Furthermore, data from Figure 11 and Figure 12 suggest that the correlation between surface fractal dimension and mechanical properties is weaker for concrete with a higher *w*/*b* ratio of 0.50 than for a lower ratio of 0.40, owing to a higher total porosity and reduced impact of capillary pores.

## 4. Conclusions

The addition of CSWs or BFs can effectively increase the compressive strength of concrete, but their excessive content will reduce the compressive strength of concrete. The addition of CSWs can effectively improve the dynamic elastic modulus of concrete, whereas the effect of BFs on the dynamic elastic modulus of concrete is not as significant as that of CSWs.

The incorporation of MCSWs can effectively enhance both the compressive strength and dynamic elastic modulus of concrete, and the improvement is more significant than that of CSWs. The enhancement effect of MCSWs on the compressive strength of concrete is greater than on its dynamic elastic modulus.

Optimal quantities of CSWs, MCSWs, and BFs contribute to a reduction in porosity and both median and average pore sizes, culminating in an improved pore structure within the concrete. This optimization leads to a decrease in the proportion of macropores and capillary pores, an increased presence of transition pores, and a negligible effect on the proportion of gel pores.

CSWs principally influence the complexity of the transitional and gel pore regions, while BFs affect the air pore region. Ink-bottle pores are predominantly located in capillary and transitional regions, with the highest concentration in capillary pores, and the surface fractal dimension generally exceeds three, implying non-fractal behavior. CSWs and their modified form impede ink-bottle pore formation, whereas BFs augment them.

Appropriate amounts of CSWs and MCSWs have been shown to increase the *D*_s3_ of concrete. While BFs can elevate both *D*_s1_ and *D*_s3_, their effect on *D*_s3_ is less significant compared to CSWs. Interestingly, CSWs, MCSWs, and BFs have a minimal impact on *D*_s4_, indicating a nuanced influence on the concrete’s microstructure. There exists a strong correlation between the compressive strength or dynamic modulus of elasticity of concrete and its surface fractal dimensions *D*_s1_ and *D*_s3_, typically characterized by a binomial power function.

## Figures and Tables

**Table 1:** Basic performance indicators of CSW.

Density (g/cm^3^)	Length (μm)	Diameter (μm)	Melting Point (°C)	Tensile Strength (GPa)	Modulus of Elasticity (GPa)	Mohs Scale	Whiteness (%)
2.69	30~150	1~4	1450	20.5	178	3~4	≥92

**Table 2:** Basic performance indicators of BF.

Diameter (μm)	Length (mm)	Density (g/cm^3^)	Modulus of Elasticity (GPa)	Tensile Strength (GPa)
20	12	2.65	250	82~87

**Table 3:** Concrete mix ratios.

No.	Water (kg/m^3^)	Cement (kg/m^3^)		Coarse Aggregate (kg/m^3^)		Fine Aggregate (kg/m^3^)		CSW (kg/m^3^)	SHMCSW (kg/m^3^)	BF (kg/m^3^)
		*w*/*b* = 0.40	*w*/*b* = 0.50	*w*/*b* = 0.40	*w*/*b* = 0.50	*w*/*b* = 0.40	*w*/*b* = 0.50			
OC	185.0	462.5	370.0	1225.7	1288.6	576.8	606.4	0	0	0
CC1	185.0	461.0	368.5	1225.7	1288.6	576.8	606.4	1.5	0	0
CC2	185.0	459.5	367.0	1225.7	1288.6	576.8	606.4	3.0	0	0
CC3	185.0	458.0	365.5	1225.7	1288.6	576.8	606.4	4.5	0	0
CC4	185.0	456.5	364.0	1225.7	1288.6	576.8	606.4	6.0	0	0
SCC	185.0	458.0	365.5	1225.7	1288.6	576.8	606.4	0	4.5	0
BF1	185.0	461.0	368.5	1225.7	1288.6	576.8	606.4	0	0	1.5
BF2	185.0	459.5	367.0	1225.7	1288.6	576.8	606.4	0	0	3.0
BF3	185.0	458.0	365.5	1225.7	1288.6	576.8	606.4	0	0	4.5
BF4	185.0	456.5	364.0	1225.7	1288.6	576.8	606.4	0	0	6.0

**Table 4:** Microstructural parameters of CSW and BF concrete based on MIP.

No.	Porosity (%)		Median Pore Diameter (nm)		Average Pore Diameter (nm)	
	*w*/*b* = 0.40	*w*/*b* = 0.50	*w*/*b* = 0.40	*w*/*b* = 0.50	*w*/*b* = 0.40	*w*/*b* = 0.50
OC	10.06	15.21	69.3	64.7	35.2	34.7
CC1	9.23	11.73	62.8	55.1	33.0	33.9
CC2	7.14	8.25	53.3	50.3	32.8	33.7
CC3	8.98	11.12	62.4	52.5	35.0	35.1
CC4	9.01	14.10	65.6	63.0	43.9	35.7
SCC	6.40	10.80	58.3	51.3	34.3	32.8
BFC1	12.61	14.99	65.2	62.8	34.0	32.3
BFC2	8.09	10.56	60.9	62.0	32.6	32.0
BFC3	11.62	16.18	70.9	54.5	41.1	30.2
BFC4	13.48	16.95	71.3	77.9	43.1	40.2

**Table 5:** Ink-bottle pore volume and hysteresis loop area of CSW and BF concrete.

	Δ$V_{1}^{\mathrm{ink}}$ (10^−3^ mL/g)		Δ$V_{2}^{\mathrm{ink}}$ (10^−3^ mL/g)		Δ$V_{3}^{\mathrm{ink}}$ (10^−3^ mL/g)		Δ$V_{4}^{\mathrm{ink}}$ (10^−3^ mL/g)		S	
*w*/*b*	0.40	0.50	0.40	0.50	0.40	0.50	0.40	0.50	0.40	0.50
OC	1.724	3.917	11.138	16.327	5.201	13.431	0	0	213.83	321.68
CC1	1.411	1.902	12.369	12.907	7.315	9.310	0	0	197.54	270.15
CC2	1.012	2.702	7.670	7.488	5.937	4.949	0	0	124.11	158.76
CC3	1.285	2.723	13.763	11.936	6.088	6.627	0	0	167.11	236.79
CC4	1.540	1.495	9.692	13.057	2.698	13.410	0	0	215.53	325.71
SCC	0.751	1.405	8.405	12.032	2.602	11.239	0	0	122.38	195.93
BFC1	1.468	1.741	13.431	16.680	8.781	13.642	0	0	323.11	363.23
BFC2	1.238	1.494	8.868	10.045	3.425	7.095	0	0	205.34	228.95
BFC3	4.708	2.574	11.370	17.289	5.847	16.203	0	0	253.66	376.53
BFC4	1.460	3.582	14.432	19.968	7.962	11.074	0	0	366.56	437.93

## Data Availability

The raw data supporting the conclusions of this article will be made available by the authors on request.

## List of Abbreviations

Nomenclature		Acronyms	
$\sum V_{n}^{\mathrm{in}}$	the cumulative volume of mercury intrusion	BF	basalt fiber
$V_{n}^{\mathrm{ink}}$	the ink-bottle pore volume	CSW	calcium sulfate whisker
$\Delta V_{n}^{\mathrm{ink}}$	ink-bottle pore volumes across various pore sizes	MCSW	modified calcium sulfate whisker
S	the hysteresis loop area	SHMP	sodium hexametaphosphate
$D_{\mathrm{sn}}$	the surface fractal dimension across various pore sizes	CSH	calcium silicate hydrate
$f_{\mathrm{cu}}$	compressive strength	MIP	mercury intrusion porosimetry
$E_{d}$	dynamic elastic modulus	SEM	scanning electron microscopy
